# From Flora to Threat: Septic Emboli Due to Corynebacterium pseudodiphtheriticum in a Patient With Relapsed Acute Myeloid Leukemia

**DOI:** 10.7759/cureus.93602

**Published:** 2025-09-30

**Authors:** Alan Wang, John Greene

**Affiliations:** 1 Osteopathic Medicine, Nova Southeastern University Dr. Kiran C. Patel College of Osteopathic Medicine, Clearwater, USA; 2 Infectious Diseases, Moffitt Cancer Center, Tampa, USA

**Keywords:** corynebacterium pseudodiphtheriticum, immunocompromised, opportunistic infection, septic emboli, treatment

## Abstract

*Corynebacterium pseudodiphtheriticum* (*C. pseudodiphtheriticum*) is typically a benign inhabitant of the upper respiratory tract but has recently emerged as a potential opportunistic pathogen in immunocompromised individuals. We report a rare case of disseminated *C. pseudodiphtheriticum* infection in a 60-year-old man with relapsed acute myeloid leukemia (AML) undergoing chemotherapy. The patient developed fever, hypotension, and a diffuse papular rash in the setting of profound neutropenia. Blood cultures from central and peripheral lines grew *C. pseudodiphtheriticum*, and computed tomography (CT) revealed multiple bilateral pulmonary nodules with ground-glass and tree-in-bud opacities, suggestive of septic emboli. Despite the organism’s frequent dismissal as a contaminant, the clinical picture, imaging, and response to therapy supported its role as the causative pathogen. Following the administration of broad-spectrum antibiotics, removal of the peripherally inserted central catheter (PICC) line, and a course of oral linezolid, the patient experienced rapid clinical and radiographic improvement. This case highlights the pathogenic potential of *C. pseudodiphtheriticum*. It underscores the need to recognize it as a true pathogen in neutropenic patients, particularly those with indwelling catheters and signs of embolic disease.

## Introduction

Septic embolism is a serious and potentially life-threatening condition in which infected thrombi or microbial aggregates travel through the bloodstream and lodge in distant vessels. This can lead to vascular blockage, tissue ischemia, and localized infections or abscesses [1]. Common sites affected include the lungs, brain, skin, and retina. These emboli typically cause both vascular obstruction and localized infection, leading to ischemia, inflammation, and tissue damage [1]. Risk factors for septic emboli include right-sided infective endocarditis, septic thrombophlebitis, intravenous drug use, long-term indwelling catheters, hematologic malignancies, chemotherapy-induced immunosuppression, and prolonged hospitalization [1,2].

Pulmonary involvement, known as septic pulmonary embolism, typically presents with fever, pleuritic chest pain, cough, and dyspnea [3]. CT imaging often reveals bilateral peripheral nodules that may cavitate or display characteristic patterns such as “feeding vessel” signs or “tree-in-bud” opacities [1-3]. In disseminated cases, cutaneous involvement may also occur, with lesions manifesting as non-pruritic erythematous or violaceous papules and nodules, sometimes progressing to pustules or necrosis, particularly in immunocompromised hosts [1]. While *Staphylococcus aureus *(*S. aureus*), *Streptococcus* species, *Enterococcus*, and certain Gram-negative rods are classically associated with septic emboli, an expanding array of atypical pathogens, such as *Corynebacterium pseudodiphtheriticum* (*C. pseudodiphtheriticum*), have been implicated in vulnerable populations. Although rare, cases of *C. pseudodiphtheriticum* endocarditis leading to embolic complications have been described in the literature [4]. However, to our knowledge, no prior reports have documented *C. pseudodiphtheriticum* as a direct cause of septic emboli in the absence of endocarditis.

*C. pseudodiphtheriticum* is a catalase- and urease-positive, non-diphtherial Gram-positive bacillus commonly found in the upper respiratory tract [4,5]. It has been shown to exert probiotic effects by competing with potential pathogens such as *S. aureus* and *Moraxella catarrhalis*, reducing their adherence to epithelial surfaces and promoting microbial homeostasis [4]. However, emerging literature suggests that *C. pseudodiphtheriticum* may transition from a commensal to a true pathogen under specific conditions, particularly in immunocompromised patients with chronic lung disease, malignancy, HIV, cystic fibrosis, or those undergoing prolonged corticosteroid therapy or endotracheal intubation [6].

In these susceptible populations, *C. pseudodiphtheriticum* has been implicated in a wide array of infections, including pneumonia, tracheobronchitis, lung abscess, endocarditis, bacteremia, keratitis, urinary tract infections, discitis, and cutaneous lesions, particularly in association with indwelling medical devices such as catheters [4,7]. Cases of infection involving the peritoneal cavity, bone, and joints have also been reported [8]. Mortality from *C. pseudodiphtheriticum* infections may be substantial in immunocompromised hosts, with some studies reporting rates as high as 23% [8]. Notably, older male patients appear to be disproportionately affected [7].

In this report, we describe a rare case of disseminated *C. pseudodiphtheriticum* infection presenting with both pulmonary and cutaneous septic emboli in a neutropenic patient undergoing chemotherapy for relapsed acute myeloid leukemia (AML). The isolate was pan-susceptible to all tested antibiotics, and the patient experienced rapid clinical and radiologic improvement following catheter removal and appropriate antimicrobial therapy. This case highlights the need to recognize *C. pseudodiphtheriticum* as a true pathogen in high-risk patients, rather than simply a benign commensal.

## Case presentation

In January 2022, a 60-year-old male with relapsed AML was admitted for reinduction chemotherapy with cladribine, cytarabine, and venetoclax. He was initially diagnosed with AML in December 2019 and underwent induction chemotherapy with a standard regimen of cytarabine for seven days and an anthracycline for three days (“7+3”), followed by four cycles of high-dose cytarabine (HiDAC) as consolidation therapy and two cycles of decitabine maintenance. He had previously deferred hematopoietic stem cell transplantation (HSCT) during his first remission due to comorbidities and opted to pursue clinical trial options at the time of relapse.

**Figure 1:**
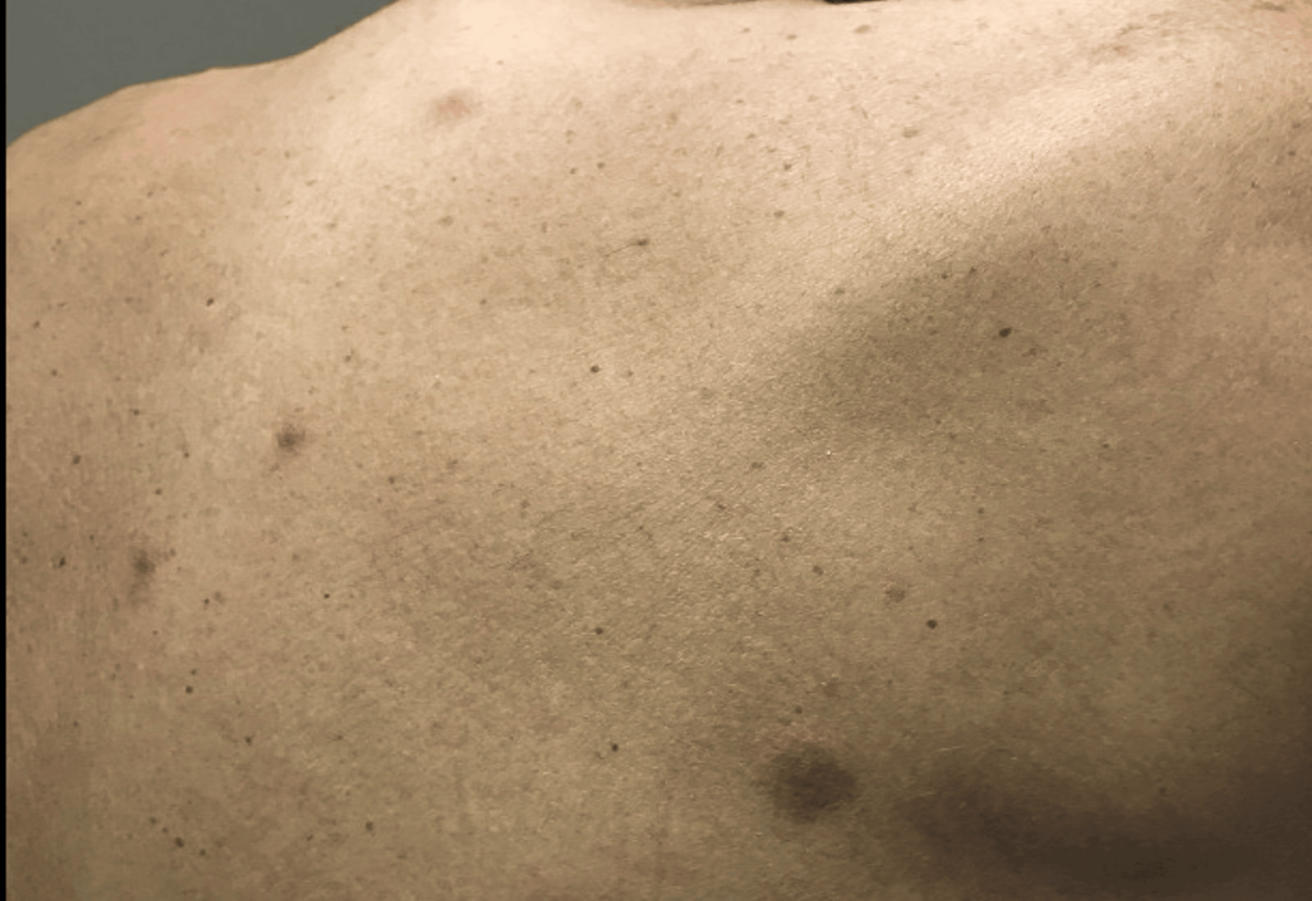
Scattered non-pruritic papules on the patient’s back are consistent with cutaneous septic emboli due to C. pseudodiphtheriticum.

On day 15 of chemotherapy (January 25, 2022), he developed a low-grade fever. Over the following 48 hours, he progressed to higher fevers (maximum temperature 101.9°F), hypotension (blood pressure 94/54 mmHg), and chills. He also developed a diffuse, non-pruritic papular rash consisting of numerous 1-cm lesions over the trunk and bilateral extremities (Figure 1). Laboratory studies revealed profound pancytopenia: white blood cell count 0.05 k/μL, hemoglobin 7.4 g/dL, and platelets 19 k/μL. The patient’s pertinent laboratory results are summarized in Table 1.

**Table 1:** Pertinent laboratory findings on hospital Day 15

Laboratory Test	Result	Reference Range
White blood cell count	0.05 k/μL	4.0–11.0 k/μL
Hemoglobin	7.4 g/dL	13.5–17.5 g/dL (male)
Platelet Count	19 k/μL	150–400 k/μL

Blood cultures drawn from both PICC and peripheral lines yielded gram-positive rods, later identified as *C. pseudodiphtheriticum*. Antibiotic susceptibility testing demonstrated pan-susceptibility to all tested agents, as shown in Table 2.

**Table 2:** Antibiotic susceptibility profile of Corynebacterium pseudodiphtheriticum isolated from blood cultures. MDIL: minimum dilution inhibitory level; MINT: minimum inhibitory concentration interpretation; MIC: minimum inhibitory concentration; S: susceptible

Corynebacterium pseudodiphtheriticum	MDIL (μg/mL)	MINT
Ceftriaxone	0.25	S
Gentamicin	2	S
Linezolid	0.5	S
Meropenem	0.016	S
Penicillin	0.03	S
Vancomycin	0.5	S

**Figure 2:**
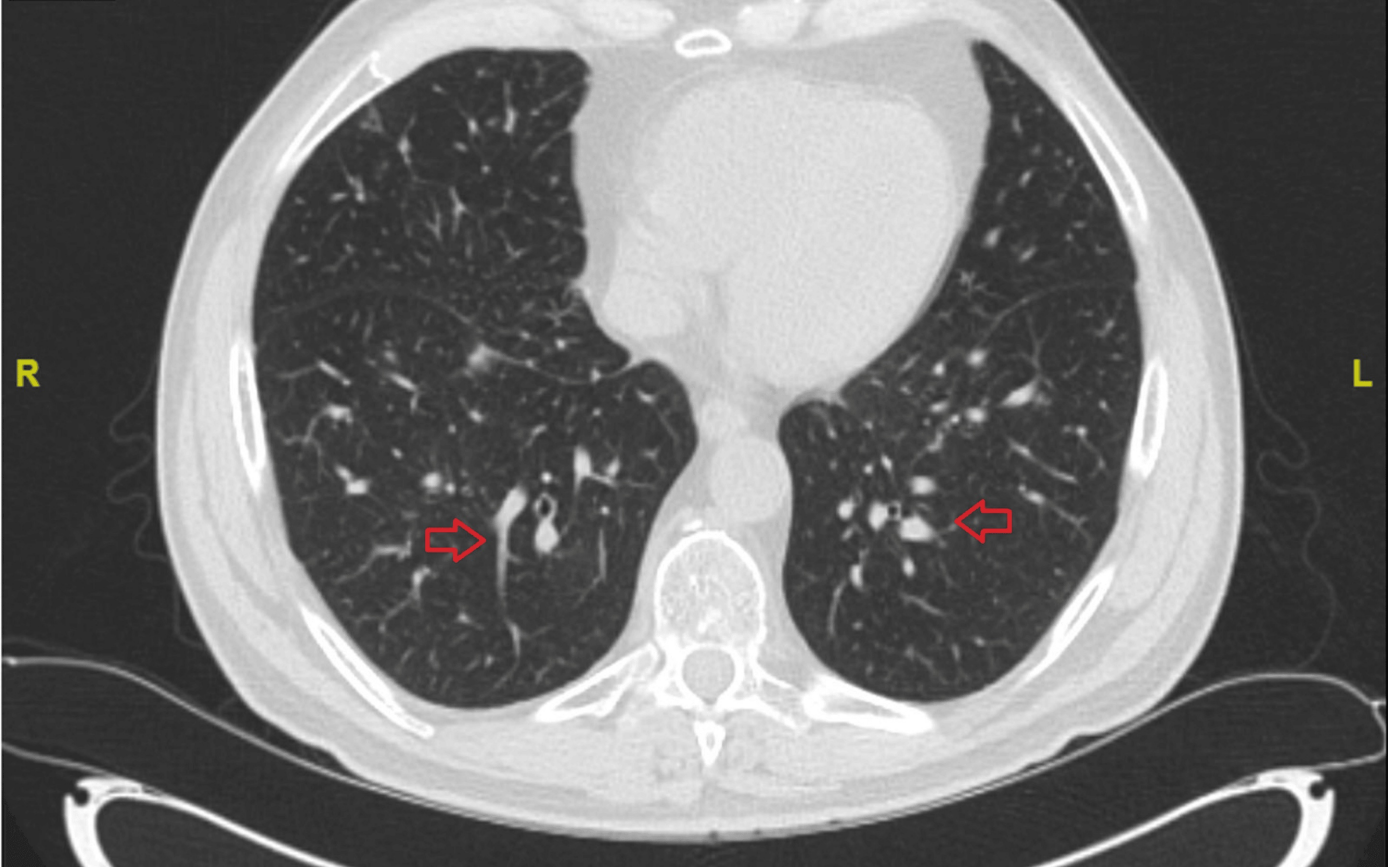
Axial chest CT without contrast showing bilateral peripheral nodular opacities with a tree-in-bud pattern, consistent with septic pulmonary emboli in an immunocompromised patient with Corynebacterium pseudodiphtheriticum bacteremia.

A non-contrast CT scan of the chest demonstrated interval development of multifocal ground-glass and small nodular opacities throughout both lungs, including tree-in-bud patterns in some regions, findings concerning for septic emboli or other infectious/inflammatory processes (Figure 2). A transthoracic echocardiogram (TTE) was performed and showed no valvular vegetations or structural abnormalities, effectively ruling out endocarditis.

The patient had initially been on levofloxacin for neutropenic prophylaxis; however, following the onset of fever, hypotension, and a new papular rash, broad-spectrum antimicrobial therapy was escalated to include vancomycin, cefepime, micafungin, ciprofloxacin, and acyclovir. Due to persistent fever and progression of cutaneous lesions, gentamicin and rifampin were later added for enhanced gram-positive and intracellular coverage. The central venous catheter was removed for source control.

A dermatologic evaluation was pursued due to concern for cutaneous septic emboli. Punch biopsy of a right lower extremity lesion revealed dermal purpura without histologic evidence of vasculitis or emboli; fungal and Grocott's Methenamine Silver (GMS) stains were negative. Nonetheless, the overall clinical and radiologic picture was consistent with disseminated infection secondary to *C. pseudodiphtheriticum* bacteremia.

On February 2 (Day 23), the patient remained afebrile but persistently neutropenic (absolute neutrophil count: 310/μL). He was discharged on February 3, 2022, on a regimen of oral linezolid (600 mg twice daily for 14 days), ciprofloxacin, acyclovir, fluconazole, and ongoing venetoclax. Repeat chest imaging on February 23 showed a decrease in size and density of pulmonary nodules, and follow-up CT on April 4 demonstrated near-complete resolution of prior lesions without evidence of new infection. The patient subsequently underwent matched unrelated donor allogeneic HSCT on April 28, 2022. He remains alive today and in clinical remission, with pulmonary and cutaneous findings fully resolved by approximately three months after infection onset. A chronological summary of key laboratory values, clinical events, diagnostics, and interventions is shown in Table 3.

**Table 3:** Timeline of key laboratory values, clinical events, and management CT: computed tomography; PICC: peripherally inserted central catheter; HSCT: hematopoietic stem cell transplantation; ANC: absolute neutrophil count; WBC: white blood cell

Date	Hospital Day	Clinical Events/Interventions	WBC (k/µL)	ANC (cells/µL)
January 10	Day 0	Admission for reinduction chemotherapy	-	-
January 25	Day 15	Fever, hypotension, rash; labs show pancytopenia	0.05	30
January 26	Day 16	Blood cultures positive for gram-positive rods; started on broad-spectrum antibiotics	-	-
January 27	Day 17	CT chest: bilateral nodules with tree-in-bud appearance; PICC line removed	-	-
January 28	Day 18	Skin biopsy performed; C. pseudodiphtheriticum identified	0.06	50
February 3	Day 24	Discharged on oral linezolid and other prophylactics	0.08	310
February 23	Day 45	Repeat chest CT: improvement of pulmonary nodules	0.9	620
April 4	Day 86	Follow-up chest CT: near-complete resolution	3.5	1500
April 28	Day 100	Underwent allogeneic HSCT	-	-

## Discussion

This case adds to the growing recognition of *C. pseudodiphtheriticum* as an opportunistic pathogen capable of causing disseminated infection in immunocompromised individuals. Although traditionally regarded as a non-pathogenic commensal of the upper respiratory tract, recent studies have increasingly implicated this organism in clinically significant infections, including lower respiratory tract disease, bloodstream infections, and endocarditis, especially in patients with hematologic malignancies, critical illness, or indwelling medical devices [6]. Among Gram-positive rods (GPRs), dissemination with septic embolization is more commonly attributed to *Corynebacterium jeikeium*, *Corynebacterium striatum*, and *Bacillus cereus*; thus, a case of septic emboli due to *C. pseudodiphtheriticum* is exceedingly rare and underscores the emerging pathogenic potential of this organism.

In our patient, several compounding risk factors likely contributed to the transition from colonization to invasive disease: profound neutropenia from chemotherapy, immunosuppressive therapy, and a PICC line. These factors, coupled with *C. pseudodiphtheriticum*'s biofilm-forming capabilities, likely facilitated bloodstream invasion and septic embolization to the lungs and skin [9].

*C. pseudodiphtheriticum* has been shown to form biofilms on abiotic surfaces, such as polyurethane catheter materials, and to bind to fibrinogen and fibronectin, enhancing its ability to adhere to host tissues and medical devices [9-11]. In vitro studies also demonstrate its ability to invade and persist within epithelial cells, evade immune clearance, and alter host actin cytoskeleton dynamics, features shared with other virulent opportunistic pathogens [4,9,12].

Importantly, as of August 2025, a comprehensive literature search using the terms "*Corynebacterium pseudodiphtheriticum*" AND "septic emboli" across PubMed, EMBASE, and CINAHL yielded no previously reported cases of septic embolization caused by this organism in the absence of endocarditis. While a few isolated cases of *C. pseudodiphtheriticum* endocarditis have been associated with embolic complications, there are no published reports describing septic emboli arising from primary bacteremia without cardiac involvement, nor any cases specifically reporting cutaneous septic emboli. In our patient, TTE revealed no valvular vegetations, suggesting that hematogenous dissemination likely occurred directly from bloodstream infection. This distinction underscores the novelty of our case and highlights a potentially underrecognized pathogenic mechanism. Given the patient’s immunocompromised state, prolonged neutropenia, and presence of an indwelling catheter, our findings support the emerging recognition that *C. pseudodiphtheriticum*, under the right conditions, can act as a true invasive pathogen capable of causing both pulmonary and cutaneous septic embolization.

These findings are especially relevant in the context of septic pulmonary embolism, a complication increasingly recognized in cancer patients with infected vascular access devices [1,2]. While *S. aureus* and *Streptococcus* species remain the most commonly implicated pathogens, *C. pseudodiphtheriticum* should be considered in the differential diagnosis when bacteremia is accompanied by compatible imaging and clinical features. In our case, the patient developed fever, hypotension, and cutaneous papules, concurrent with positive blood cultures for *C. pseudodiphtheriticum* and CT findings of peripheral ground-glass and nodular opacities with a tree-in-bud appearance, a classic radiographic hallmark of septic emboli [1].

A major challenge in managing *C. pseudodiphtheriticum* infections is the frequent misclassification of this organism as a contaminant, particularly when isolated only once or alongside other organisms in polymicrobial cultures [4]. This stems from its common presence in the upper respiratory tract and on the skin, which often leads clinicians to dismiss its clinical significance, especially in patients without overt signs of infection. However, mounting evidence has demonstrated that in the presence of host immunosuppression, prolonged hospitalization, prior antibiotic exposure, and indwelling devices, this organism can act as a true pathogen capable of causing severe systemic infections, including septic emboli, as seen in our patient.

The antibiotic susceptibility profile of *C. pseudodiphtheriticum* is variable. Resistance to macrolides is well-documented, with multidrug-resistant strains also being reported in Brazil and France [4,13,14]. Macrolide resistance is attributed to bacterial erm-class methylase genes, which modify ribosomal RNA and inactivate the drug’s mechanism of action [15]. However, the organism generally remains susceptible to linezolid, vancomycin, penicillin, and beta-lactams [5,16].

Management of septic emboli hinges on prolonged targeted antibiotic therapy and source control. In cases involving *C. pseudodiphtheriticum*, some experts recommend at least six weeks of antibiotic treatment, particularly due to the organism’s biofilm-forming ability and potential for endovascular involvement [17]. In our patient, fevers and progression of cutaneous lesions persisted despite empiric broad-spectrum therapy until the central venous catheter was removed and rifampin and gentamicin were added. Following this, the patient defervesced within 48 hours. The patient was later transitioned to oral linezolid with continued clinical improvement. Repeat imaging within three weeks showed partial resolution of pulmonary nodules, and by three months, near-complete resolution was observed. Skin lesions also resolved within several weeks of therapy. Anticoagulation is generally not indicated in the management of septic emboli unless the thromboembolic risk clearly outweighs bleeding concerns.

The largest systematic review to date on *C. pseudodiphtheriticum* (1983-2023) documents a growing number of infections, particularly among hospitalized and immunocompromised populations, across a wide array of organ systems, including the lungs, heart, urinary tract, skin, and catheter-related infections [4]. To illustrate the expanding clinical spectrum and the context in which these infections occur, Table 4 presents selected reported cases along with their associated risk factors. This growing recognition is also supported by advancements in diagnostic microbiology, most notably the adoption of 16S rRNA gene sequencing and MALDI-TOF mass spectrometry, which have greatly improved the accuracy of *Corynebacterium* species identification [18].

**Table 4:** Reported infections caused by Corynebacterium pseudodiphtheriticum PICC: peripherally inserted central catheter; COPD: chronic obstructive pulmonary disease; HIV: human immunodeficiency virus

Infection Type	Reported Risk Factors	References
Bacteremia	Indwelling catheters, malignancy, neutropenia	[4,6,7]
Endocarditis ± emboli	Native or prosthetic valves, PICC	[4,17]
Pneumonia	Intubation, chronic lung disease (COPD, cystic fibrosis), HIV	[6,12]
Cutaneous lesions (wound)	Immunosuppression, hematologic malignancy	[4,8]
Discitis/osteomyelitis	Hematogenous spread, post-surgical	[4]
Keratitis	Contact lenses, ocular trauma	[4]
Urinary tract infection	Catheterization, older male age	[4,8]
Pulmonary and cutaneous septic emboli (non-endocarditis)	Neutropenia, indwelling catheter, hematologic malignancy	This case

This case highlights the critical importance of early catheter removal, the integration of microbiologic findings with clinical context, and the need for heightened awareness of atypical pathogens such as *C. pseudodiphtheriticum* in immunocompromised hosts. Although our patient’s skin biopsy did not demonstrate histologic evidence of septic emboli, the constellation of symptoms, imaging findings, and clinical response supported a diagnosis of disseminated infection. Opportunistic organisms, particularly those with biofilm-forming capacity and the ability to persist intracellularly, can cause significant embolic disease in vulnerable hosts, especially those with prolonged neutropenia and compromised mucosal barriers. Clinicians should maintain a broad differential for septic emboli in cancer patients and not dismiss unusual organisms as contaminants, as early recognition and timely intervention are essential for favorable outcomes.

## Conclusions

This case illustrates an unusual presentation of pan-susceptible *C. pseudodiphtheriticum* bacteremia with secondary septic pulmonary and cutaneous emboli in a neutropenic patient undergoing chemotherapy for relapsed AML. It highlights the evolving pathogenic role of this organism, especially among those with weakened immune systems. It underscores the importance of recognizing *C. pseudodiphtheriticum* as a potential true pathogen rather than a routine contaminant. Prompt identification, appropriate antimicrobial therapy, and catheter removal led to favorable outcomes in our patient. Clinicians should maintain a high index of suspicion for septic pulmonary emboli in cancer patients with new lung lesions and bloodstream infections, even when the organism identified is traditionally regarded as low-virulence or commensal.
